# The DYD-RCT protocol: an on-line randomised controlled trial of an interactive computer-based intervention compared with a standard information website to reduce alcohol consumption among hazardous drinkers

**DOI:** 10.1186/1471-2458-7-306

**Published:** 2007-10-26

**Authors:** Elizabeth Murray, Jim McCambridge, Zarnie Khadjesari, Ian R White, Simon G Thompson, Christine Godfrey, Stuart Linke, Paul Wallace

**Affiliations:** 1 E-health Unit, Department of Primary Care and Population Sciences, University College London, Highgate Hill, London N19 5LW, UK; 2 Centre for Research on Drugs & Health Behaviour, London School of Hygiene & Tropical Medicine, Keppel Street, London WC1E 7HT, UK; 3 MRC Biostatistics Unit, Institute of Public Health, Forvie Site, Robinson Way, Cambridge CB2 0SR, UK; 4 Department of Health Sciences, Seebohm Rowntree Building, University of York, Heslington, York YO10 5DD, UK

## Abstract

**Background:**

Excessive alcohol consumption is a significant public health problem throughout the world. Although there are a range of effective interventions to help heavy drinkers reduce their alcohol consumption, these have little proven population-level impact. Researchers internationally are looking at the potential of Internet interventions in this area.

**Methods/Design:**

In a two-arm randomised controlled trial, an on-line psychologically enhanced interactive computer-based intervention is compared with a flat, text-based information web-site. Recruitment, consent, randomisation and data collection are all on-line. The primary outcome is total past-week alcohol consumption; secondary outcomes include hazardous or harmful drinking, dependence, harm caused by alcohol, and mental health. A health economic analysis is included.

**Discussion:**

This trial will provide information on the effectiveness and cost-effectiveness of an on-line intervention to help heavy drinkers drink less.

**Trial registration:**

International Standard Randomised Controlled Trial Number Register ISRCTN31070347

## Background

### Alcohol consumption

Excessive alcohol consumption is a significant public health problem throughout the world. Regular heavy alcohol consumption and binge drinking are associated with physical problems, mental health problems, antisocial behaviour, violence, accidents, suicides, injuries, road traffic accidents, unsafe sexual behaviour, underperformance at work or school, and crime. People who drink too much can be described as hazardous or harmful drinkers, although there is considerable overlap between these two categories. Hazardous drinkers are those whose alcohol consumption puts them at risk of physical, mental or social harm, while harmful drinkers are those who are already experiencing harm as a result of alcohol consumption. It has been estimated that 26% of the population of England (38% of men and 16% of women aged 16 – 64) drink hazardously or harmfully, equating to approximately 7.1 million people in England alone [1], and costing the NHS £1.7bn p.a. [2]. Alcohol misuse is a problem throughout Europe and comes third after smoking and high blood pressure as the most significant risk factor for ill health in the European Union [3]. In the US in 2002 – 03, 23% of the population reported binge drinking in the past month [4]. The health care costs of alcohol misuse in the US have been estimated at $26.3 billion for 1998, but these costs are a small proportion of the total estimated cost of alcohol misuse estimated at $184.6 billion [5].

### Interventions to promote safer drinking

There are a range of effective interventions for alcohol problems [6]. These have been categorised into brief interventions, specialist treatments, and less intensive treatment interventions which fall between the first two [6]. Within this categorisation, brief interventions are delivered to people who are not seeking help for an alcohol problem from specialist alcohol services – that is people who have been identified as drinking hazardously or harmfully through a screening or case-finding approach. They are carried out in general community settings and are delivered by non-specialist staff, such as general practitioners or other primary healthcare staff, hospital doctors and nurses, social workers, probation officers and other non-specialist professionals. There is a large body of evidence confirming the effectiveness of such interventions in reducing alcohol consumption and alcohol-related harm in hazardous drinkers [7-10].

Similar interventions, delivered to a help-seeking population, have been classified as less intensive interventions. Such interventions typically extend from 1 – 4 sessions, and can be delivered by specialist alcohol workers, or generalists with a special interest in treatment of alcohol problems [6]. These interventions have also been found to be effective in treatment of alcohol problems, and probably as effective as more intensive treatment [6,9]. Finally, there are a very wide range of specialist treatments for alcohol problems, usually reserved for people with more severe problems, including those with co-morbidities. Effective interventions target the drinker's motivation to reduce their alcohol consumption and their ability to control alcohol consumption. Table 1 outlines the main interventions with empirical support for their effectiveness in problem drinkers.

**Table 1:** Components of Effective Interventions for Alcohol Misuse.

**Component**	**Description**
Motivational Interviewing	A client-centred, directive method for enhancing intrinsic motivation to change by exploring and resolving ambivalence^31^.
Motivational Enhancement Therapy	An adaptation of Motivational Interviewing. Usually consists of 4 sessions, often with the user's significant other^6^.
Cognitive Behavioural Therapy	Seeks to identify and modify maladaptive cognitions, prescribe specific coping strategies, teach coping behaviours through instruction, modelling, directed practice and feedback, and teaches specific problem-solving strategies^6^.
Behavioural Self-control Training	Also called self-management training. Usually includes setting limits for drinking; self-monitoring of alcohol consumption; methods to control the rate of drinking; drink-refusal skills training; self-reward systems for successful behaviours; analysis of antecedents to excessive drinking; training alternative behaviours to drinking to cope with high-risk relapse situations^6^.
Social behaviour and network therapy	Based on the premise that people with serious drinking problems need to develop positive social network support for change. Components include identifying and contacting network members and engaging them in treatment^6^.
Coping and social skills training	Enables the user to live a fulfilling life without excessive drinking. Often combined with assertiveness training and/or communication skills training^6^.
Behaviour contracting	The therapist negotiates agreement between the service user and their significant other to a system of mutual expectations and obligations^6^.
Conjoint marital therapy	Suitable for socially stable alcohol misusers with moderate dependence/alcohol problems and an intact partnership^6^.

Despite the evidence supporting use of brief and less intensive interventions in people with alcohol problems, recent data suggest that fewer than 1 in 18 people with an alcohol misuse disorder in the UK have access to appropriate treatment [1].

### Interactive computer-based interventions

Interactive computer-based interventions (ICBI) (also called Interactive Health Communication Applications) provide information and one or more of decision support, behaviour change support and emotional support for health issues [11,12]. Early examples of computer-based interventions were delivered on CD-ROM or Interactive Video Discs. More recently, the Internet has been the favoured method of delivery, partly for reasons of reach (e.g. 61% of the UK population in 2005 had Internet access at home [13]), and partly as Internet delivery allows rapid central updating as new research becomes available. With the convergence of digital technology, such interventions can be transferred to digital television or mobile telephone platforms. This will have a significant effect on the "digital divide" (the gap between those with regular access to digital and information technology and those without) as these three delivery systems (digital television, mobile telephones and the Internet) between them achieve nearly universal population penetration.

Interactive computer-based interventions have great potential as a method for achieving behaviour change, as they can include [11]:

• Multiple self-assessment tools;

• Exercises to promote reflection, and consider the benefits and disbenefits of change;

• Tailored information, provided in response to self-assessment questionnaires, with the ability to store almost limitless quantities of information, but only provide relevant information in comprehensible, accessible "bite-size" chunks;

• The ability for an individual to set personalised goals, and document sources of support, barriers to change, and methods for overcoming these barriers;

• On-line social support, both in terms of electronic support groups, and in the form of personal stories from people in similar situations who have achieved change.

They are also popular with users, as they are convenient (can be used at any time of day or night), anonymous and confidential. They can be revisited as often as wanted, so have the potential not only to promote behaviour change, but also to prevent relapse. A Cochrane systematic review of interactive computer-based interventions for people with long-term conditions found that their use was associated with improved knowledge, self-efficacy, perceived social support, health behaviours and clinical outcomes [12].

The main costs associated with interactive computer-based interventions occur during the development phase. Once completed, there are relatively few costs associated with use, and these do not increase with the number of users. Hence such interventions have the potential of being an extremely cost-effective method of achieving behaviour change for large numbers of people simultaneously.

### Interactive computer-based interventions for alcohol

Efforts to explore the potential of ICBI for hazardous or harmful drinkers are underway internationally [14-19]. A variety of pilot studies support the potential of this approach. The Drinker's Check-up is a non-web-based, stand-alone computer-based programme which takes about 90 minutes to complete. Data on 35 participants suggested that four weeks after use of the programme, participants in the intervention group had reduced their average number of drinks per day in the assessment period by about 50%, while those in the waiting-list (control) group had reduced by about 27%. Longer-term comparison was not possible, as the waiting-list group had access to the intervention after 4 weeks [20]. A web-based screening and brief intervention programme (Check Your Drinking, now renamed the Alcohol Help Centre) has been developed in Canada [15], and modified for use in Finland [18]. This programme screens users for alcohol consumption (using the 10 item Alcohol Use Disorders Identification Test (AUDIT))[21], and provides normative feedback. Users of this programme reduced their mean number of drinks in a typical week from 19.10 at baseline to 13.83 at the three-month follow-up [15,18]. A randomised controlled trial (RCT) of a brief web-based screening and brief intervention for university students undertaken in New Zealand demonstrated a 26% reduction in alcohol consumption in the intervention group at 6 weeks, although this difference was no longer present at 6 months. This intervention took a mean of 11.2 minutes to complete, and contained assessment (in the form of a 14-day retrospective drinking diary) and feedback [16]. An RCT in Holland of an on-line self help intervention demonstrated reduced mean weekly alcohol consumption in the intervention group compared to the control group at 6 and 12 months of follow-up [19].

Linke et al developed Down Your Drink (DYD) in the UK in 2000 [14]. This interactive, web-based intervention was originally structured as a set of six consecutive intervention modules, designed to be accessed by users at weekly intervals. The programme was based on the stages of change model [22], and contained adaptations of components common in brief interventions, including cognitive behavioural therapy and relapse prevention. A naturalistic cohort study of 10,000 users of the programme demonstrated a clinically highly significant reduction in alcohol-related harm amongst those who completed the 6 – week programme, with between one-third and one-half decrease (improvement) in scores on measures of alcohol dependence (Short Alcohol Dependency Data [23]), alcohol related harm (Alcohol Problems Questionnaire [24]) and mental health (Clinical Outcomes in Routine Evaluation – Outcome Measure [25]). However attrition was extremely high, with only 16.5% completing all six modules [26].

## Methods/Design

### Aim

The aim of the trial is to determine the effectiveness and cost-effectiveness of an on-line, psychologically enhanced, interactive computer-based intervention (DYD) in reducing alcohol consumption amongst members of the public at risk of harm from alcohol, when compared with a flat, text-based information website.

The objectives of the trial are to:

• Determine the effectiveness of DYD in enabling users to reduce their total alcohol consumption;

• Determine the effectiveness of DYD in reducing alcohol-related harm in users;

• Determine the costs associated with development and use of DYD;

• Determine the cost-effectiveness of DYD as a public health intervention.

### Design

This is an on-line, two-arm randomised controlled trial, comparing web-based health information on the potential harms of alcohol only, with web-based information plus interactive features designed to enhance user motivation and self-efficacy in modifying their alcohol consumption. We hypothesise that the interactive, psychologically enhanced, components known to be effective in alcohol interventions, including motivational enhancement, behavioural self-control and cognitive behavioural therapy will be more effective in achieving behaviour change amongst users than the flat, text-based health information alone, as information by itself is unlikely to be effective in achieving behaviour change [27]. For the purposes of the trial, both the intervention and the comparator comprise one web-site, known as Down Your Drink (DYD). People in the intervention group are given access to the entire site, including all the interactive theoretically driven components, while those in the control group only have access to flat, text-based information. For the duration of the trial, access to DYD is limited to those who consent to participate in the trial and provide base-line data.

The entire trial is conducted on-line, including recruitment, consent, randomisation and data collection. Ethical approval has been obtained from the University College London ethics committee.

### Setting and participants

Participants are Internet users who have found the DYD site on the World Wide Web. DYD is hosted on Alcohol Concern's website and accessed by a link from the homepage. Alcohol Concern is the major UK charity concerned with providing education, help, and resources for people concerned about alcohol consumption.

### Recruitment

People who access the DYD site are invited to take a screening test, consisting of three questions on the frequency of alcohol consumption, average number of drinks per consumption, and frequency of drinking six or more drinks on one occasion (the AUDIT-C[28,29]).

Adults aged 18 or over who score 5 or more on the AUDIT-C, indicating hazardous or harmful drinking, are invited to participate in the DYD trial. The nature of the recruitment procedures and the trial itself require users to have Internet access. People who declare themselves unable to understand written English, or unwilling to complete follow-up questionnaires are excluded. Those interested in participating have to access sequentially a series of information pages, culminating in a consent form. Once the consent form has been completed, the user is asked for their e-mail address, and a self-selected user ID and password; submission of these data triggers an automatic e-mail from the DYD site, providing them with a link to the rest of the baseline data collection pages.

### Randomisation

Randomisation occurs in two stages. The first randomisation occurs after completion of consent and core baseline data. At this point, participants are randomised to receive one of the four secondary outcome measures (see section 3.6). Once all baseline data have been completed, participants are randomised to either the intervention or the control group. This second randomisation marks the trial entry point. Both randomisation procedures are done by centrally-allocated computer-generated random numbers.

### Intervention

The original DYD website was significantly updated and expanded for this trial. The content and presentation of the site were adapted in response to user feedback on the original site, advances in the brief interventions and alcohol treatment literature, and literature on user requirements of interactive web-based interventions [30]. A process of iterative user feedback collected both on-line and in focus groups was used to improve the site in terms of navigation and content. The finalised psychologically enhanced intervention contains three phases: a phase aimed at helping the user assess their current drinking and reach a decision about whether or not they should change their drinking, a phase aimed at acting on their decision, and a phase aiming to prevent and deal with lapses and relapses. Users are able to view the phases in any order they wish or view recommended selections of pages and can view the site whenever, and as often, as they choose. Highly dependent drinkers are advised of the dangers of stopping drinking suddenly, and advised to seek additional medical help at relevant points throughout the site.

Phase 1, called "It's up to you", is designed to help users consider their drinking and reach a decision about whether or not they would like to change anything about their drinking. This phase is based on the principles of Motivational Interviewing [31], and contains four levels. The first level encourages the user to consider their drinking, including what is good and less good about it, how much the user drinks compared to others, and the harms and benefits of alcohol. The second level develops this further, with more self-assessment exercises for the user to reflect on their alcohol consumption and the role alcohol plays in their life. The third level deals with dilemmas in decision-making, while the fourth crystallises the decision-making process, leading users to make a decision and plan a specific change. All users are encouraged to prepare for change by using these Phase 1 resources, with a view to arriving at a high quality decision.

Phase 2 is called "Making the change", and is designed to help users implement the decision they made in Phase 1. Support in this phase and Phase 3 are based on behavioural self-control techniques, adapted for on-line use. Users are encouraged to consider high risk occasions for drinking heavily and other threats to personal change goals. Users may either reduce their alcohol consumption or choose complete abstinence; guidance on both goals is provided.

Phase 3 is called "Keeping on Track", and provides information and help for users who have made a change in their alcohol consumption. Level 1 in this phase is designed to help prevent lapses becoming relapses, emphasising that to lapse is normal, but need not knock the user off track, while level 2 focuses on preventing lapses.

A key feature of the intervention is the use of "e-tools" and automated communication between the user and the site. The most prominent "e-tool" is the drinking episode diary. This enables the user to regularly record the details of their drinking and extract data that they can use for self analysis of their drinking behaviour. The other main "e-tool" is the "thinking drinking log" which records all of the user's free text responses to questions throughout the programme and makes them available for the user to review. The communications module includes e-mail reminders to log on to the site, news and automated "drinking tips" which are sent to the user via e-mail.

The comparator site uses a similar graphical design and style to the intervention site and contains a great deal of evidence-based information about the physical, mental and social harms caused by alcohol. However, there are no tools to enhance individual motivation or monitor alcohol consumption, no self-assessment materials, and content is minimally informed by cognitive-behavioural perspectives.

### Outcome measures

Baseline data to be collected include:

• basic demographic details (completion of the age and gender fields is mandatory while provision of off-line contact details is encouraged but not mandatory);

• highest level of educational attainment;

• total past week alcohol consumption (using the TOT-AL, a specially designed and validated on-line instrument to determine past week alcohol consumption) [32];

• two single item measures of intention to change drinking and self-efficacy in achieving this change;

• the EQ-5D [33] (a five item health status questionnaire devised for health economic analysis);

• and one of 4 secondary outcome measures, measuring different domains of alcohol-related harm.

One of the many methodological challenges in behavioural intervention research is possible reactivity of assessment – that simply completing assessment measures induces change. This issue is particularly acute in brief alcohol intervention research, given the similarities between research assessment and intervention content. Assessment effects have been studied and effect sizes quantified in this field [34,35]. For this reason, we opted to randomly allocate participants to one of 4 secondary outcome measures to minimise the assessment burden. These measures are:

• Alcohol Use Disorders Identification Test (AUDIT) [21] (measuring hazardous or harmful drinking)

• Leeds Dependence Questionnaire [36] (measuring dependence on alcohol)

• The ten-item Clinical Outcomes in Routine Evaluation (CORE-10) [37] (measuring mental health problems, not specific to alcohol)

• Alcohol Problem Questionnaire [24] (measuring problems related to excess alcohol consumption).

We will also explore possible effects on more specific aspects of alcohol consumption, such as binge drinking, derived from the primary outcome measure.

### Data collection

Data are collected at baseline (prior to randomisation), three months and 12 months. All data collection is done on-line, through the trial website, accessed via a hot-link in an e-mail. The trial website is separate from the intervention and comparator websites, and has a completely different "feel", engendered by use of different colours, different style of presentation, and different content. The trial website is designed to appeal to user altruism, by emphasising the experimental nature of the two different forms of DYD, our ignorance as to which is better, and the importance of follow-up data from all participants to inform future decisions.

A further methodological challenge is the expected high rate of attrition from follow-up. The original cohort study (section 1.4) had a follow-up rate of 16.5% at 6 weeks. We have incorporated a number of features designed to improve this, including:

• The recruitment and consent procedures emphasise the importance of follow-up;

• The trial website appeals to user altruism, and aims to engender a feeling of being part of an important scientific project;

• Three e-mail reminders at seven day intervals to non-responders;

• The use of an appropriate incentive;

• A final (4^th^) reminder to non-responders with a request to complete a simplified version of the primary outcome measure only.

### Analyses

#### Sample size calculation

The principal end-point is at three months. The primary outcome is the last week's total alcohol consumption in units of alcohol. The observed mean reduction in weekly alcohol consumption in the cohort study was 35% in men (from 39 units in week 1 to 25 units in week 6) and 17% in women (from 24 to 20 units) [26]. A 20% reduction, irrespective of initial level, is typical of non-internet brief interventions [9]. The standard deviation of weekly alcohol consumption in the cohort study was slightly less than the mean, in both men and women, and at both baseline and follow-up. Making a conservative assumption that the standard deviation is equal to the mean, 430 subjects providing follow-up data at three months in each arm would give us 90% power at the 5% significance level to detect a 20% reduction in the past week's alcohol consumption.

Follow-up rates in the cohort study were only 16.5% at 6 weeks [26], but we expect the measures designed to improve retention will achieve follow-up on one-third of participants at 3 months. Hence we need to randomise 2,580 subjects (1290 per group). The duration of the trial recruitment phase will be 12 months.

#### Analyses

The primary analysis will be the mean difference in changes from baseline in all outcome variables between the two groups at three months, using all available results but without imputation of missing data. We will perform an adjusted analysis using regression of outcome on randomised group, adjusted for baseline value of outcome and other variables (e.g gender, education). Secondary analyses will include all randomised individuals (1) by assuming that non-responders have no change in their alcohol consumption, (2) by imputing missing outcomes using other outcomes and website use data, and (3) by considering plausible arm-specific differences between responders and non-responders [38]. Non-response bias will be explored in terms of trend in outcome across number of reminders needed for response [39], and predictors of non-response in terms of baseline characteristics and web usage [40].

Subgroup analyses will be performed by assessing interactions of the primary outcome with a maximum of three pre-determined factors (education, gender, and baseline alcohol consumption). Additional exploratory analyses will be undertaken for the secondary outcomes.

Additional analyses will include descriptive statistics to characterise participants in terms of demographics, alcohol consumption, intention and self-efficacy to change, EQ-5D, and secondary outcome measures; CONSORT diagram [41] of the flow of participants through the trial, and proportion who completed each registration stage to trial entry; and use of intervention and comparator web-sites.

### Health economic analysis

The primary health economic analysis will assess the incremental cost-effectiveness of the full DYD compared to the provision of flat information only from an NHS perspective. The costs of developing the two websites will be derived from actual cost records from the project. The main outcome for the economic analysis will be Quality Adjusted Life Years. This will be obtained from responses to the EQ-5D [33] questionnaire at baseline, three months and 12 months and population values of health states using the area under the curve method [42]. Sensitivity analysis will be conducted using different methods of imputation of missing data and bootstrapping methods will be used to analyse the uncertainty around the calculated incremental cost-effectiveness ratios and cost-acceptability curves.

A secondary analysis will be performed to model the longer term consequences of changes in alcohol consumption on a range of alcohol related consequences. These analyses will be from a societal perspective. This model will be based on primary data from previous randomised controlled trials and observational data combined with existing systematic reviews. Consequences considered will include future impacts on quantity and quality of life, health and other public service resource use and wider social effects. The costs of interventions will also include an estimate of participants' time engaged in the intervention.

## Discussion

This on-line randomised controlled trial has the potential to address two key issues. Its primary purpose is to determine whether website interactivity and psychological enhancement adds significantly to the potential impact on users' health and on related outcomes of a behavioural health related website of this kind. This is an important issue, given the rapid rise in health related websites and their increasing use by both members of the public and health care professionals.

The trial will additionally provide potentially valuable information about whether an entirely-on-line trial methodology of this kind can be used to assess effectiveness and cost-effectiveness. This on-line trial of an on-line intervention is fraught with methodological challenges. Traditional methodological approaches for randomised controlled trials may not be appropriate for this different research environment, and the problems commonly faced in off-line trials (e.g. recruitment) may not be problems in on-line ones.

We see the major challenges as: retention (follow-up at 3 and 12 months); randomisation and contamination (the degree to which participants attempt/succeed in re-registering with alternative identities to obtain access to both the intervention and comparator sites); validity of outcome data (both because all data are self-reported, including age and gender, and because paper-and-pencil measures must be transferred on-line); and finally, the construction of a credible and ethically-acceptable comparator site to control for non-specific intervention factors. We have undertaken extensive piloting to address these issues, which will be reported separately.

## Abbreviations

AUDIT Alcohol Use Disorders Identification Test

AUDIT-C Alcohol Use Disorders Identification Test – C

CD-ROM Compact Disc Read Only Media

CONSORT Consolidated Standards of Reporting Trials

CORE Clinical Outcomes in Routine Evaluation

DYD Down Your Drink

EQ-5D EuroQol Five Dimensional

ICBI Interactive Computer Based Interventions

NHS National Health Service

RCT Randomised Controlled Trial

TOT-AL measure of TOTal ALcohol consumption over past 7 days

UK United Kingdom

US United States

## Competing interests

The author(s) declare that they have no competing interests.

## Authors' contributions

All authors have had substantial intellectual contribution to this protocol. IW and ST have led on the analytical strategy, CG led on the health economics, SL and JM led on developing the intervention and comparator websites, and EM led on the overall methodological development. ZK is the Research Fellow on the project and PW is Principal Investigator. EM wrote the first and final drafts of the protocol; all authors have contributed to the drafting process, and all authors have read and approved the final manuscript.

## Pre-publication history

The pre-publication history for this paper can be accessed here:


